# Tight focusing field of cylindrical vector beams based on cascaded low-refractive index metamaterials

**DOI:** 10.1515/nanoph-2023-0261

**Published:** 2023-08-07

**Authors:** Lan Ke, Chenxia Li, Simeng Zhang, Bo Fang, Ying Tang, Zhi Hong, Xufeng Jing

**Affiliations:** Institute of Optoelectronic Technology and the Centre for THz Research, China Jiliang University, Hangzhou 314423, China; College of Metrology & Measurement Engineering, China Jiliang University, Hangzhou 310018, China; Centre for THz Research, China Jiliang University, Hangzhou 310018, China

**Keywords:** 3D printing, cascade, metamaterial, metasurface, terahertz, vector light

## Abstract

Using low-refractive-index metamaterials, we design a transmission-type radial-angular cylindrical vector beam generator. A high numerical aperture lens is constructed using an asymmetric meta-grating structure. The metamaterial vector beam generator and the meta-grating lens are physically cascaded to obtain the tight focusing characteristics of the vector light field. The vector beam generator module and the meta-lens module are prepared by 3D printing technology, and the near-field test has been carried out on the samples in the terahertz band. Using the physical cascading method, two modules are cascaded to construct a vector beam tight focusing device, and the focusing electric field distribution test has been carried out. The use of 3D printing technology for sample preparation further reduces the manufacturing difficulty and production cost, and ensures the realization of its design function on the basis of miniaturization and light weight, which provides the possibility for the research of tight focusing field regulation in the terahertz band.

## 1 Introduction

Polarization, as an important characteristic of light, has always been a hot spot in optical research, especially the research on vector beams with non-uniform polarization distribution has aroused great interest of researchers [1–4]. The particular solutions of the most basic cylindrical vector beams (radial and angular vector beams) have very important applications in modern optics because of their unique polarization distribution [5, 6]. The tight focusing operation of the cylindrical vector beam can be applied in the fields of micromachining, optical imaging, information storage, and optical tweezers technology [7–9].

Recently, K. S. Youngworth et al. theoretically analyzed and studied the tight focus distribution characteristics of radial-angular vector beams [10]. Typically, a cylindrical vector beam tightly focused field is generated directly from the light source, or the beam is pre-converted using a spatial light modulator and then focused using a high numerical aperture lens [11, 12]. In the terahertz wave band, the traditional devices required to generate the vector tight-focus field are complex, numerous and expensive, which is very unfavorable for the actual research of the terahertz vector beam tight-focus field [13–17]. The method of cascading metamaterials and metasurfaces and producing them through 3D printing can well solve the above problems [18–25].

Metamaterials are artificial materials with special properties, which can effectively and flexibly control electromagnetic waves, and metasurfaces are two-dimensional manifestations of metamaterials [26–31]. Combining the two can regulate the electromagnetic wave more carefully, and meanwhile modulate the polarization characteristics, amplitude, and phase of the electromagnetic wave [32]. The rise of 3D printing technology has opened up more possibilities for our research on metamaterials. It has tremendous advantages in terms of preparation precision, speed, cost, and customization. With the use of 3D printing, metamaterials can be designed and modified conveniently and quickly, greatly improving the efficiency of both metamaterial design and production. Additionally, 3D printing is capable of effectively reducing production costs, especially in short-term and customized production.

Here, we chose the low-refractive-index material red wax with high print accuracy and utilized simulation for preliminary design. With the use of 3D printing technology, we produced the metamaterial and metasurface modules, and then physically cascaded the two modules. In the experimental aspect, we first tested each module separately and then tested the overall cascaded system, studying its performance and vector characteristics and comparing them with the simulation results. We achieved a tightly focused field of radial and angular vector beams.

## 2 Principle and design

### 2.1 Realization principle

To realize the tightly focused field generation of radial-angular vector beams, we cascaded metamaterial arrays with metasurface lens. The first is to use the metamaterial array to flexibly control the phase and amplitude of the incident beam wavefront, and convert a pair of orthogonally polarized beam with linearly polarization into radial cylindrical vector beam and angular cylindrical vector beam, respectively.

**Figure 1:**
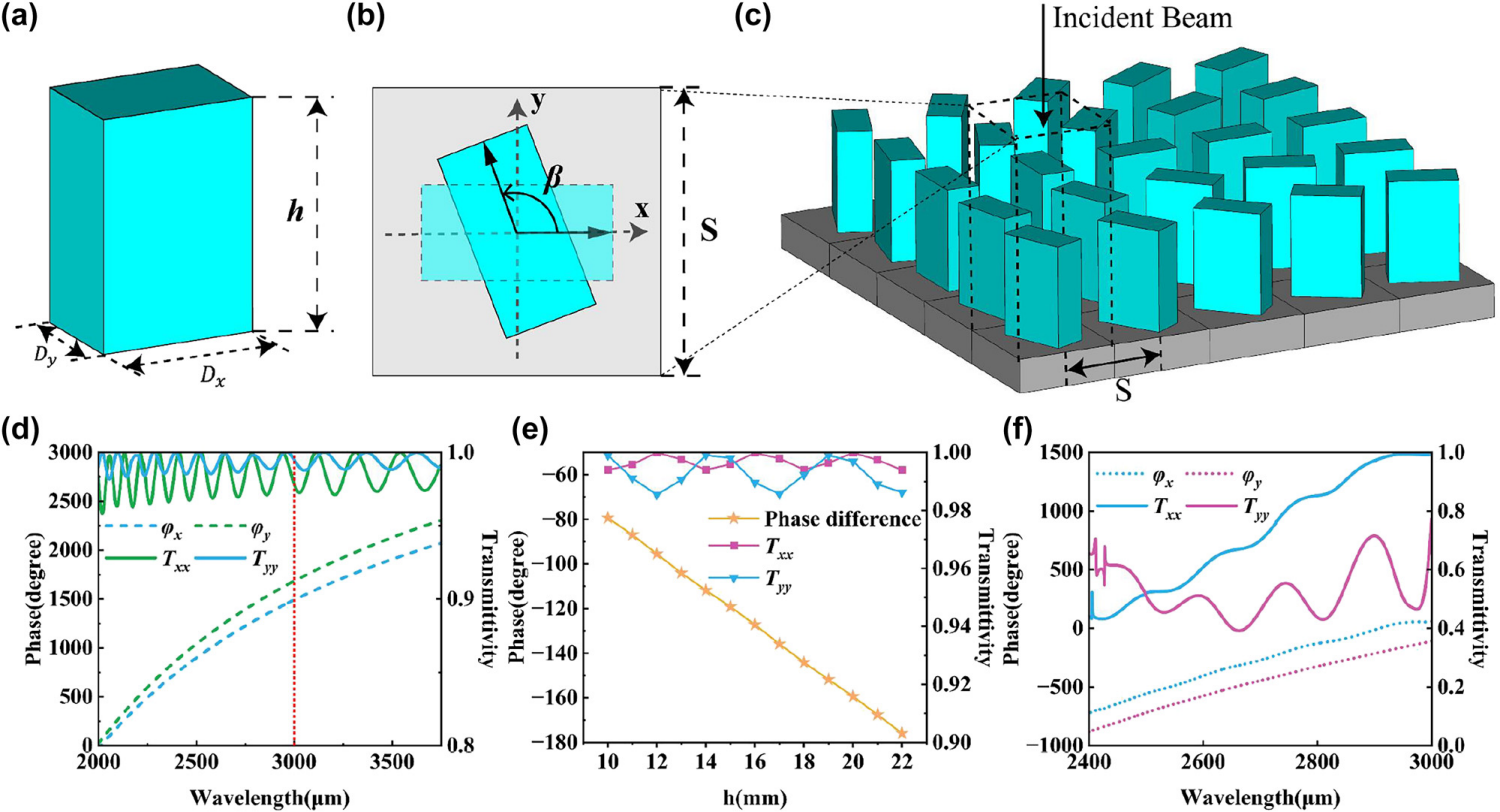
Transmission amplitude and phase characteristics of element structures. (a) Schematic diagram of metamaterial unit structure. (b) Metamaterial unit period and rotation mode. (c) Schematic diagram of metamaterial array unit distribution. (d) Transmissivity and phase parameters of the 1500 μm-period unit cells. (e) Schematic plot of the transmissivity and phase shift of the 1500 μm-period unit cells as a function of height. (f) Transmissivity and phase parameters of the 3000 μm-period unit cells.

The metamaterial array is integrated by rectangular pillar units as shown in Figure 1(a). The unit structure can be optimized and designed separately by changing the dimensions of the major axis *D*_*x*_, the minor axis *D*_*y*_, and the column height *h* to achieve precise wavefront control of the incident plane wave. The material of the unit structure is red wax resin. Metamaterial pillars can modulate the phase and polarization of incident light waves without changing the amplitude [33–41]. It can be represented by a symmetric unitary matrix *T*. Due to the properties of unitary matrices, the Jones matrix *T* can be decomposed as:

(1) $$T=V\Delta V^{T}=V\left[\begin{array}{cc} {\text{e}}^{\text{i}\varphi_{x}} & 0\\ 0 & {\text{e}}^{\text{i}\varphi_{y}} \end{array}\right]V^{T}.$$

Among them, Δ is the eigenvalue matrix. *V* is a real unitary matrix, which corresponds to the angle *β* of the in-plane geometric rotation of the unit, as shown in Figure 1(b). Since *V* ^*T*^ = *V* ^−1^, *V* ^*T*^ represents rotation −*β*. Therefore, the manipulation of metamaterial to realize the Jones matrix *T* can be thought of as rotating the electric field of the incident wave (*E*_in_) by −*β*. The components of the rotated *E*_in_ in the *x* and *y* directions are respectively endowed with phase shifts *φ*_*x*_ and *φ*_*y*_, and the rotated and phase-shifted vector is rotated by *β* angle to return to its original position. The adjustment of *φ*_*x*_ and *φ*_*y*_ can be realized by controlling the size of *D*_*x*_ and *D*_*y*_. Therefore, the conversion of cylindrical vector beams can be realized by freely selecting *φ*_*x*_, *φ*_*y,*_ and the in-plane rotation angle *β*.

In order to realize the generation from orthogonal linear polarization to radial-angular vector beams, the unit structure should be equivalent to a half-wave plate [42–48]. Initially, the unit parameters were selected based on the chosen operating wavelength of 3000 μm and the manufacturing precision of the 3D printer. To scale the unit cell at sub-wavelength scales, we first set the unit cell period S into 1.5 mm. By choosing unit cells with *D*_*x*_ = 1.2 mm and *D*_*y*_ = 0.5 mm for numerical simulation, a half-waveplate characteristic can be obtained at a column height of *h* = 22 mm, as shown in Figure 1(d). It can be observed that the unit cell exhibits good transmissivity and phase characteristics in the wavelength range from 2000 μm to 3700 μm, with an overall transmissivity of 0.96 or higher and a smooth phase curve. Figure 1(e) shows the transmissivity and phase difference curves with a constant *D*_*x*_ and *D*_*y*_, but varying column height. Under the column height variation from 10 mm to 22 mm, the transmissivity remains at a high level, and the phase difference exhibits a stable linear change. Therefore, the design can be selected according to different requirements. Based on these parameters, 3D printing was conducted. However, the excessively slender structure resulted in insufficient overall module stability, which decreased the practicality of the module. Therefore, the unit period was enlarged. Simultaneously, after enlarging the structure in a proportional manner, further parameter scanning optimization was conducted on the size of the major and minor axes to reduce the height of the column and increase its structural strength. By obtaining the current unit parameters at a 3 mm period, the height of the column was reduced from 22 mm to 16 mm, which significantly enhanced the stability and practicality of the structure. The final structure of the metamaterial unit was determined to have *D*_*x*_ = 2 mm, *D*_*y*_ = 1 mm, a column height *h* of 16 mm, and a unit period *S* of 3 mm. As shown in Figure 1(f), the transmissivity of this unit has decreased. However, in the wavelength range of 2400 μm–3000 μm, the smooth variation of the phase curve can still be ensured, and the performance of the half-wave plate can be achieved in the entire band, which can further reduce the experimental error.

Incorporate the characteristics of a half-wave plate into Equation (1), Equation (1) transforms into:

$$T=\left[\begin{array}{cc} \cos\beta & -\sin\beta \\ \sin\beta & \cos\beta \end{array}\right]\left[\begin{array}{cc} 1 & 0\\ 0 & -1 \end{array}\right]\left[\begin{array}{cc} \cos\beta & \sin\beta \\ -\sin\beta & \cos\beta \end{array}\right]$$

(2) $$=\left[\begin{array}{cc} \cos2\beta & \sin2\beta \\ \sin2\beta & -\cos2\beta \end{array}\right].$$

When *E*_in_ is *x*-polarized beam, it can be expressed as:

(3) $$T\cdot E_{\text{in}}=\left[\begin{array}{cc} \cos2\beta & \sin2\beta \\ \sin2\beta & -\cos2\beta \end{array}\right]\left[\begin{array}{c} 1\\ 0 \end{array}\right]=\left[\begin{array}{c} \cos2\beta \\ \sin2\beta \end{array}\right].$$

Similarly, when *E*_in_ is *y*-polarized beam, after *T* conversion, it can be expressed as:

$$T\cdot E_{\text{in}}=\left[\begin{array}{cc} \cos2\beta & \sin2\beta \\ \sin2\beta & -\cos2\beta \end{array}\right]\left[\begin{array}{c} 0\\ 1 \end{array}\right]=\left[\begin{array}{c} \sin2\beta \\ -\cos2\beta \end{array}\right]$$

(4) $$=\left[\begin{array}{c} \cos\left(2\beta -\frac{\pi }{2}\right)\\ \sin\left(2\beta -\frac{\pi }{2}\right) \end{array}\right].$$

According to Equation (4), we found that the conversion from *y*-polarized beam to angular vector beam can use the same metamaterial array as for *x*-polarized beam conversion. The conversion metamaterial array designed according to the polarization characteristics of radial-angular vector beams is shown in Figure 1(c). The complete metamaterial module is comprised of 19 × 19 unit cells, constructed on a square substrate with a side length of 70 mm. Sufficient space has been allocated around the substrate to enable cascading using a fixture.

The formation of a tightly focused field also requires a lens with a high numerical aperture to focus and shape the cylindrical vector beam [49]. In order to better match the cascade of metamaterial array and lens in the fabrication process, we chose to use the same material to construct metasurface lens for design and fabrication. The metasurface is easy to integrate and process, and it can make cascaded devices have miniaturization characteristics. Based on the diffraction energy distribution characteristics of asymmetric scattering mode metasurface units, we can design metasurface lens with high numerical aperture [50–57]. This scattering pattern is different from conventional phase mapping methods. The conventional phase gradient mapping method is achieved by arranging units with different phases under a certain spatial resolution to cover phase values ranging from 0 to 2π in different period of deflection. This lens can achieve efficient beam deflection with fewer unit structures.

**Figure 2:**
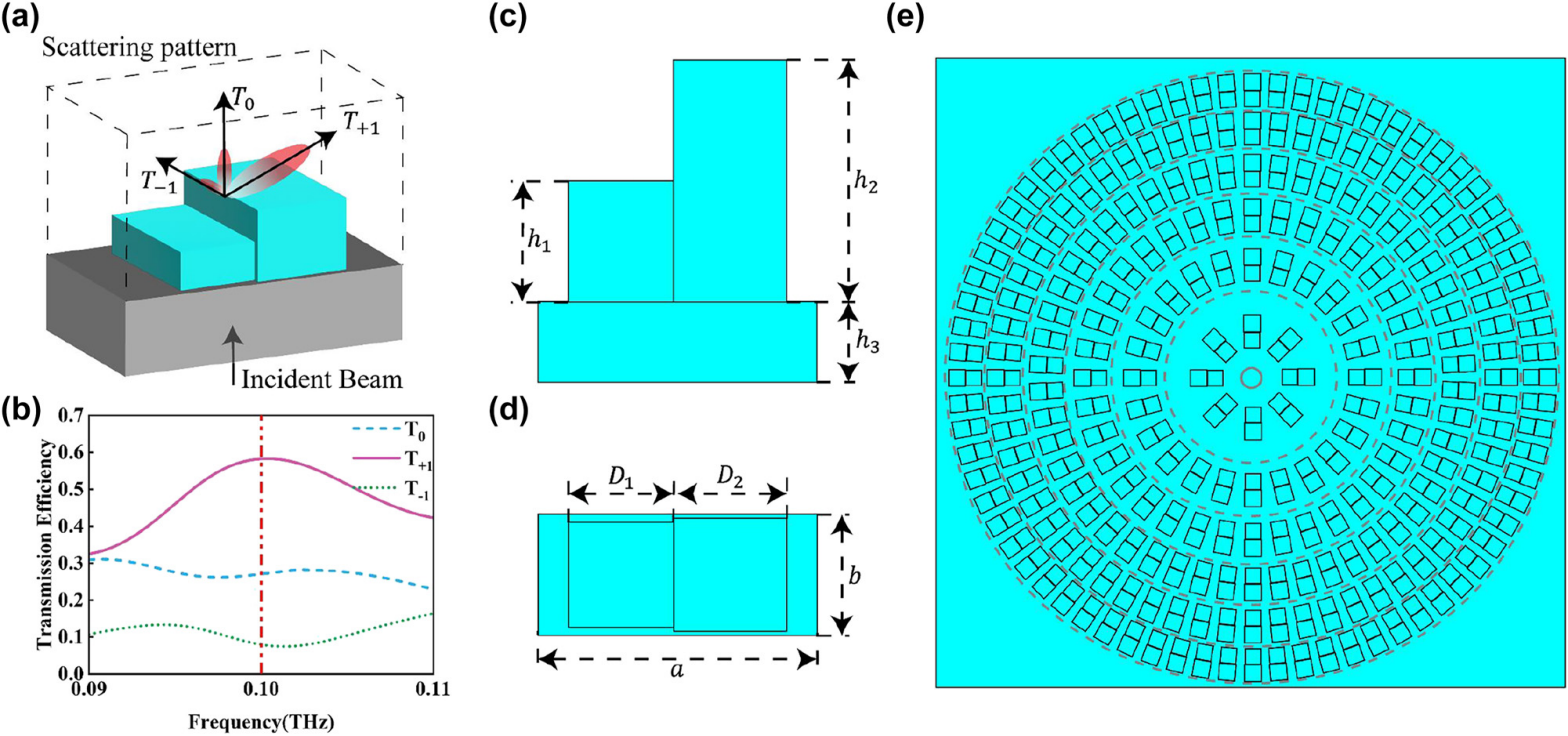
The asymmetric double column structure. (a) Schematic diagram of the diffraction energy distribution of the asymmetric double column structure. (b) The diffraction efficiency distribution under the incident mode of linearly polarized light. (c) The front view of the asymmetric double column structure. (d) The top view of the asymmetric double column structure. (e) Schematic diagram of the distribution of metasurface array units.

The working principle of the cascaded lens is shown in Figure 2(a), a rectangular unit diffracts the incident light. The unique structural design on the rectangular substrate enables the selective distribution of diffraction energy on the diffraction energy level, and concentrates as much energy as possible in the desired deflection direction (*T*_+1_), rather than *T*_0_ and *T*_−1_ order. The specific design of the unit structure is shown in Figure 2(c) and (d). An asymmetric double column structure is built on a rectangular substrate, and all structural materials are Red Wax Resin. The side lengths of the two square columns are *D*_1_ = 1.3 mm, *D*_2_ = 1.4 mm, column height is *h*_1_ = 1.5 mm, *h*_2_ = 3 mm. The long side of the rectangular substrate is the diffraction period. Optimizing *a* can adjust the deflection angle. Therefore, for a given wavelength and diffraction angle, the size of *a* is fixed. The short side *b* is a non-diffraction period, which will not affect the diffraction. Therefore, the value of *b* should be as small as possible to avoid diffraction while maintaining a high array density. The substrate thickness is *h*_3_ = 1.5 mm. The energy conversion effect of unit diffraction is shown in Figure 2(b). The energy of the *T*_+1_ order is obviously higher than that of other orders, and the *T*_−1_ order is the smallest.

Based on the feasibility of the experimental preparation, we design the lens with a numerical aperture of NA = 0.87, a focal length of *F* = 16 mm, and a maximum scattering angle of 60°. The relationship between the size of the array period and the deflection angle can be expressed as [58]:

(5) $$\delta =\sin^{-1}\left(\frac{\lambda }{a}\right).$$

Among them, *δ* is the deflection angle, and *λ* is the wavelength. At a wavelength of *λ* = 3000 μm, *α* can be calculated as 3464 μm using Equation (5). This size significantly limits the number of cells that can be accommodated, and in order to achieve the deflection effect, at least two or more cells must be accommodated within that distance range if we employ the method of phase gradient mapping. A small number of cells would greatly impact the effectiveness of the deflection. Moreover, different deflection angles are required at different positions, and a fixed-period unit structure cannot finely match the variation of *α*. The utilization of phase gradient overlay also increases the demand for the cell phase, requiring multiple different cells to achieve deflection and thus increasing the difficulty of design. Based on the diffraction energy distribution characteristics of the asymmetric scattering pattern of the metasurface element, the designed unit can deflect the beam using only a single element, with finely controlled deflection angles achieved by simply changing the size of the diffraction period.

The schematic diagram of the lens array is shown in Figure 2(e). The size of the lens is limited by the focal length, and cells on different regions of the lens are designed to produce different deflection angles corresponding to their radial positions within the lens, thereby approximating the ideal parabolic phase mapping to a piecewise linear phase mapping. The non-diffractive period is kept constant (*b* = 1.5 mm), while the diffractive period *a* is varied in order to generate the first diffractive order at the corresponding angle. The unit structure on each ring belt is integrated as much as possible under the premise of ensuring the deflection effect, so as to maximize the energy conversion efficiency.

**Figure 3:**
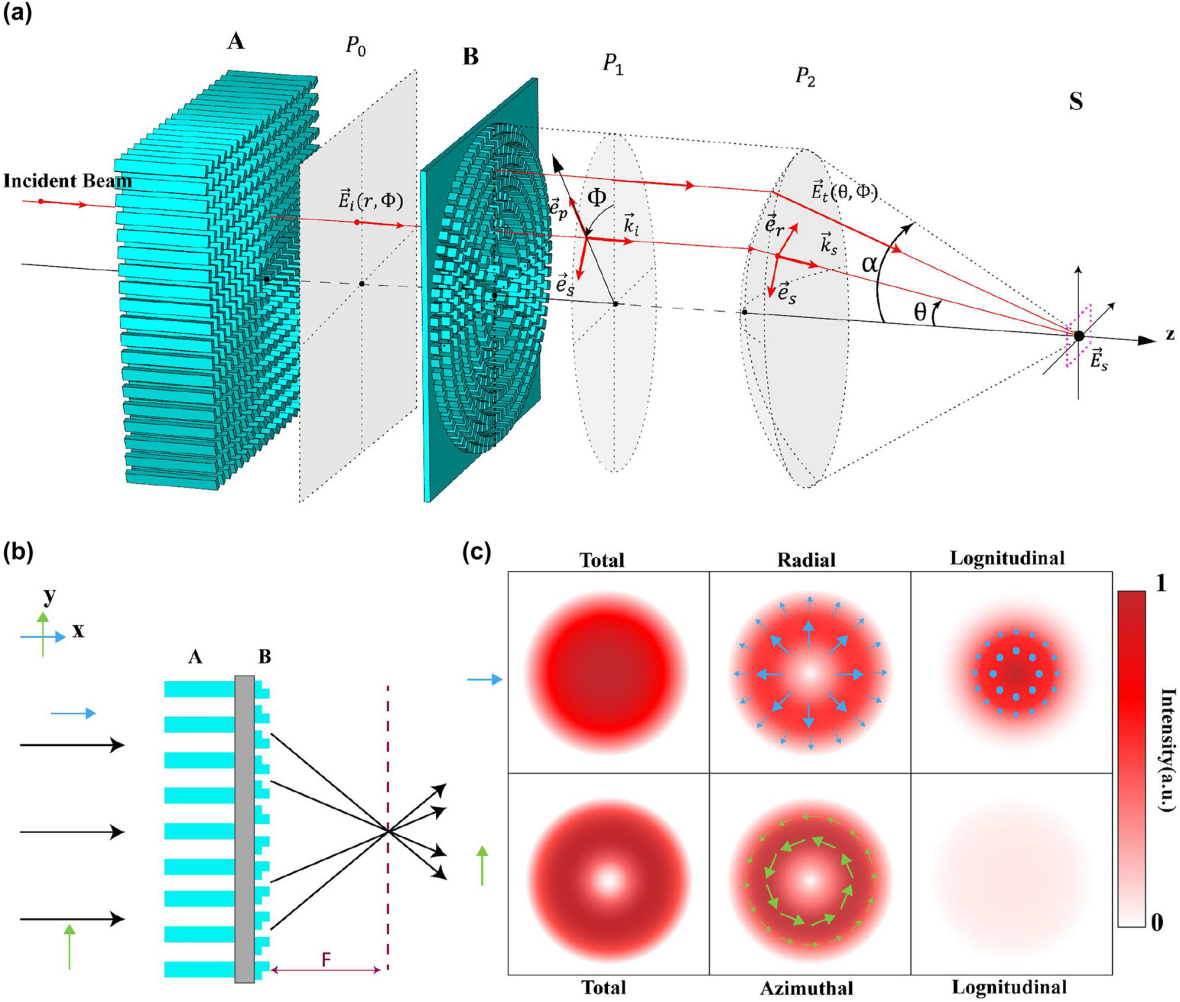
Cascading tight focusing property. (a) Schematic representation of the overall structure and function. (b) Schematic diagram of device cascading and conversion functions. (c) Theoretically derived total field intensity distribution at the focal point of the tightly focused field and light intensity distribution of each component.

The schematic diagram of the entire device is shown in Figure 3(a). Based on Richards–Wolf vector diffraction theory, the theoretical distribution of the focusing field can be calculated [59]. The incident beam is first converted into the corresponding ${\vec{E}}_{i}\left(r,\Phi \right)$ through *A* (metamaterial array). The *P*_0_ plane presents a cylindrical vector beam distribution, and then converged and shaped by *B* (metalens) to produce a tightly focusing effect. When the beam is incident on the *B* plane, *P*_1_ and *P*_2_ are used for equivalent analysis. and the incident beam is decomposed in polar coordinates, its radial component ${\vec{e}}_{p}$ and angular component ${\vec{e}}_{s}$ are, respectively:

(6) $$\vec{e_{p}}=\cos\Phi \vec{i}+\sin\Phi \vec{j}=\left[\begin{array}{c} \cos\Phi \\ \sin\Phi \\ 0 \end{array}\right]$$

(7) $$\vec{e_{s}}=\vec{k_{i}}\times \vec{e_{p}}=-\sin\Phi \vec{i}+\cos\Phi \vec{j}=\left[\begin{array}{c} -\sin\Phi \\ \cos\Phi \\ 0 \end{array}\right].$$

The essence of the transformation of light by the lens is to convert plane waves into spherical waves for convergence [60–68]. The radial components and wave vectors after spherical conversion are shown in Equations (8) and (9):

(8) $$\vec{e_{r}}=\cos\theta \left(\cos\Phi \vec{i}+\sin\Phi \vec{j}\right)+\sin\theta \vec{k}=\left[\begin{array}{c} \cos\theta \cos\Phi \\ \cos\theta \sin\Phi \\ \sin\theta \end{array}\right]$$

$$\vec{k_{s}}=\vec{e_{r}}\times \vec{e_{s}}$$

(9) $$=\cos\theta \vec{k}-\sin\theta \left(\sin\Phi \vec{j}+\cos\Phi \vec{i}\right)=\left[\begin{array}{c} -\sin\theta \cos\Phi \\ -\sin\theta \sin\Phi \\ \cos\theta \end{array}\right]$$

where *θ* is the deflection angle, and its maximum value is *α*. According to the Richards–Wolf vector diffraction theory, the light field distribution (plane *S*) near the focus is the diffraction integral of the light field on the *P*_2_ plane:

(10) $$\vec{E_{s}}=\frac{-ik}{2\pi }\underset{\Omega }{\iint }\vec{\alpha }\left(\theta ,\Phi \right)\exp\left(ik\left(\vec{k_{s}}*\vec{r}\right)\right)d\Omega .$$

Of which:

(11) $$\vec{\alpha }\left(\theta ,\Phi \right)=f\sqrt{\cos\theta }\vec{E_{t}}\left(\theta ,\Phi \right)$$

is the weight factor of the light field.

When the radial vector beam is incident, that is:

(12) $$\left[\begin{array}{c} \vec{i_{s}}\\ \vec{j_{s}}\\ \vec{k_{s}} \end{array}\right]=\left[\begin{array}{c} \cos\theta \cos\Phi \\ \cos\theta \sin\Phi \\ \sin\theta \end{array}\right]$$

We can obtain the vector diffraction integral at the focal point according to Equation (10), and simplify it by the Bessel function of the first kind, of order *n*:

(13) $$\overset{2\pi }{\underset{0}{\int }}\cos\left(n\Phi \right)\exp\left(ik\rho_{s}\sin\theta \cos\Phi \right)=2\pi i^{n}J_{n}\left(k\rho_{s}\sin\theta \right)$$

(14) $$e_{\rho }^{\left(s\right)}=k\overset{a}{\underset{0}{\int }}l_{0}\sin\theta \cos\theta \sqrt{\cos\theta }J_{1}\left(k\rho_{s}\sin\theta \right)\exp\left(ikz_{s}\cos\theta \right)d\theta$$

(15) $$e_{\Phi }^{\left(s\right)}=e_{\rho }^{\left(s\right)}\overset{2\pi }{\underset{0}{\int }}\tan\left(\Phi -\Phi_{s}\right)d\Phi =0$$

(16) $$e_{z}^{\left(s\right)}=-ik\overset{a}{\underset{0}{\int }}l_{0}\sin\theta^{2}\sqrt{\cos\theta }J_{0}\left(k\rho_{s}\sin\theta \right)\exp\left(ikz_{s}\cos\theta \right)d\theta .$$

Equation (13) is the Bessel function of the first kind, of order *n*, and Equations (13)–(15) are the vector diffraction integrals of radial component, angular component, and longitudinal component (*z* direction) in sequence.

Similarly, we can get the vector diffraction integral when the angular vector beam is incident:

(17) $$\left[\begin{array}{c} \vec{i_{s}}\\ \vec{j_{s}}\\ \vec{k_{s}} \end{array}\right]=\left[\begin{array}{c} -\sin\Phi \\ \cos\Phi \\ 0 \end{array}\right]$$

(18) $$e_{\rho }^{\left(s\right)}=0$$

(19) $$e_{\Phi }^{\left(s\right)}=k\overset{a}{\underset{0}{\int }}l_{0}\sin\theta \sqrt{\cos\theta }J_{1}\left(k\rho_{s}\sin\theta \right)\exp\left(ikz_{s}\cos\theta \right)d\theta$$

(20) $$e_{z}^{\left(s\right)}=0.$$

Establishing the relationship between the focus field and the incident field using vector diffraction integration.

The cascaded way of the whole device is shown in Figure 3(b), component A is built directly on the opposite side of the meta-lens (B) substrate. 3D printing technology can print the entire sample into an integrated shape. Choosing such a physical cascading method can make the structure more compact while ensuring the function and the overall device is more miniaturized and integrated. According to the previous vector diffraction analysis, the theoretical focus field distribution is shown in Figure 3(c). The total field distribution of the tightly focused field produced when *x*-polarized light is incident is a solid circular focus with no angular component. The radial component presents the radial vector light polarization state distribution of light intensity singularity. The optical field distribution exhibits a longitudinal component with a wavevector direction parallel to the polarization direction. When *y*-polarized light is incident, there is no radial component and longitudinal component, only the angular component exists. Its distribution presents the distribution characteristic of the polarization state of angular vector light, and the light intensity presents the distribution of the central void.

### 2.2 Simulation

Using the finite-difference time-domain method to carry out numerical simulation of the modules we designed, all modules work at 0.1 THz (wavelength *λ* is 3000 μm). Both modules of the cascaded device choose red wax resin as the construction material, and its refractive index is 1.68, which is a low refractive index material. The cascaded device is essentially a double-sided structure on a single substrate. High refractive index materials will amplify the resonance between the two-sided structures, affecting the stability and accuracy of the simulation results. This low refractive index material has a good transmission effect in the terahertz band and can obtain better conversion efficiency. In order to compare the effect of the two modules before and after cascading, we first simulate the performance of the two modules, respectively.

**Figure 4:**
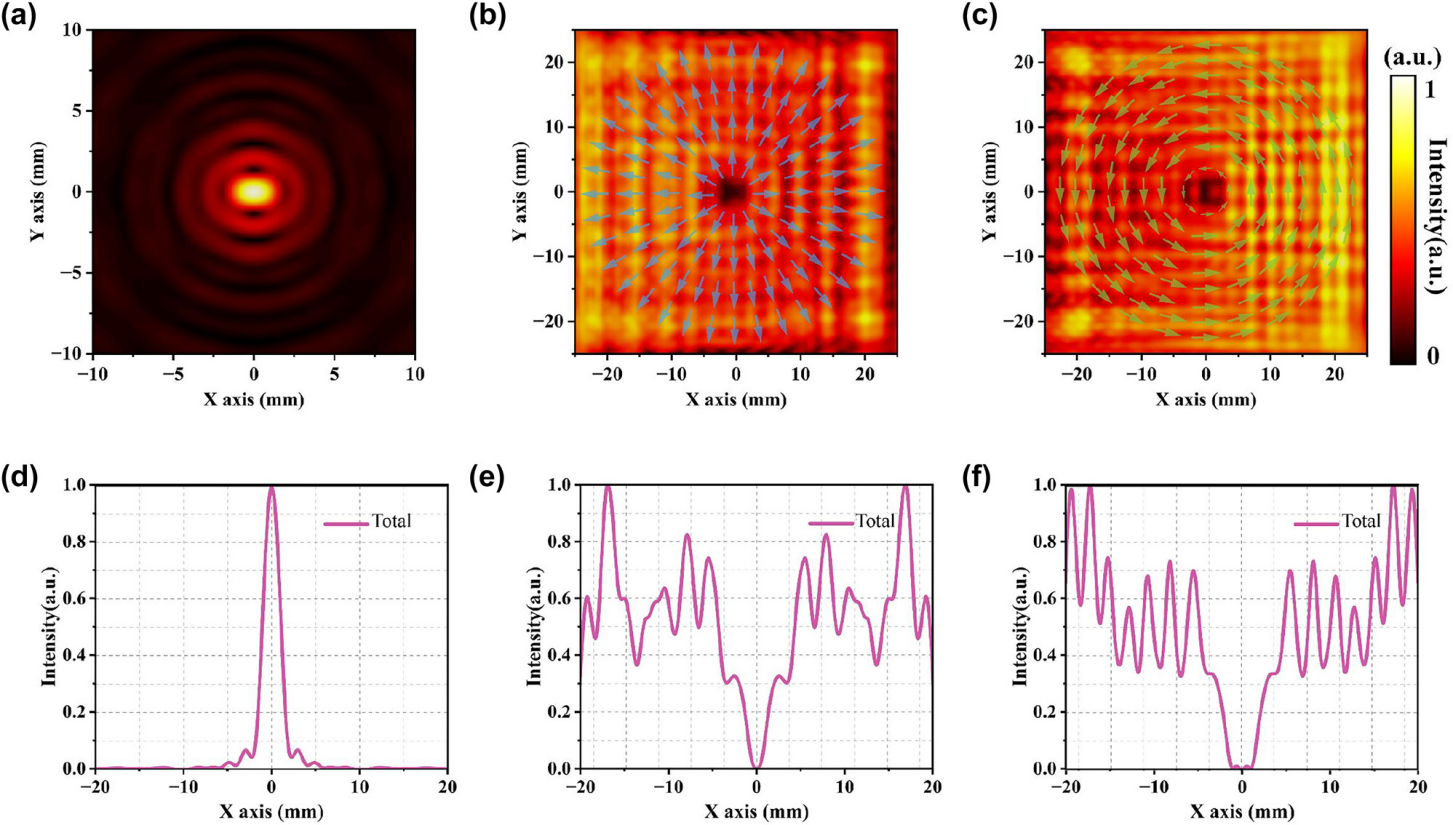
Electromagnetic field distribution characteristics. (a) The normalized intensity distribution diagram of the focal plane of the metalens. (b) The normalized intensity distribution and electric field vector distribution diagram of the *x*-polarized light emitted by the metamaterial array. (c) Normalized intensity distribution and electric field vector distribution diagram of metamaterial array converted *y*-polarized light output. (d) Normalized light intensity distribution curve along the *x*-axis at the focal plane of the meta-lens. (e) The normalized light intensity distribution curve of the radial vector light along the *y*-axis. (f) The normalized light intensity distribution curve of the angular vector light along the *y*-axis.

The simulation sampling is carried out at 16 mm in the focusing direction of the metalens, and the normalized electric field intensity distribution of the metalens is shown in Figure 4(a). Since the metalens array is a completely symmetrical structure, the conversion effect on the incident orthogonally polarized beams is consistent, and both can produce an elliptical focus. The light intensity distribution on the *x*-axis is shown in Figure 4(d). It can be seen that the focusing effect is good, and almost all the energy is concentrated at the focal point. The focal length can reach the expected design, and the light intensity distribution is similar to the Bessel distribution, except for the focal point, there are secondary light intensity rings. Figure 4(b) shows the normalized electric field intensity distribution and polarization distribution after conversion of the metamaterial array when *x*-polarized light is incident. The polarization direction of the light field diverges along the radial direction, which conforms to the polarization distribution characteristics of the radial vector light, and is still linearly polarized light without polarization conversion. The center of the light field presents a singular point of light intensity due to the uncertainty of the polarization state, and the inhomogeneity of the light intensity distribution can be clearly observed. This is because the metamaterial unit will produce an effect similar to the F–P resonant cavity when the beam is incident [69–74], which will have a certain confinement effect on the beam energy, making the final light intensity distribution closer to a discrete pixelated distribution. Figure 4(e) shows the *y*-axis light intensity distribution of the sampling plane. Similarly, when the *y*-polarized light is incident, the outgoing electric field intensity distribution (Figure 4(c)) presents a similar effect to the incident *x*-polarized light, and its polarization distribution conforms to the polarization distribution characteristics of the angular vector beam. Figure 4(f) is the *y*-axis light intensity distribution curve of the sampling plane, which clearly presents a singular point of light intensity at the center.

**Figure 5:**
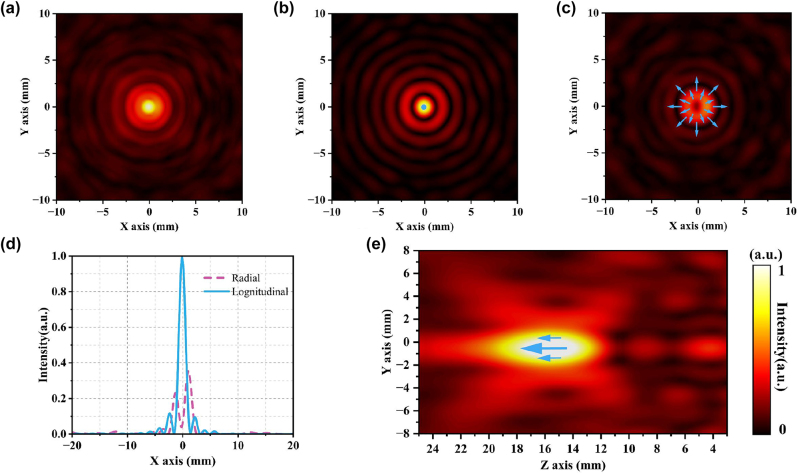
Numerical simulation results of cascaded metamaterials with *x*-polarized light incident: (a) Normalized focal plane total field intensity distribution. (b) Normalized focal plane longitudinal field distribution and the polarization distribution at the focal point. (c) Normalized focal plane radial field distribution and polarization distribution at the focal point. (d) Normalized light intensity distribution curves of the focal plane along the *x*-axis longitudinal field and radial field. (e) The side electric field intensity distribution along the *z*-axis and the focal polarization distribution.

**Figure 6:**
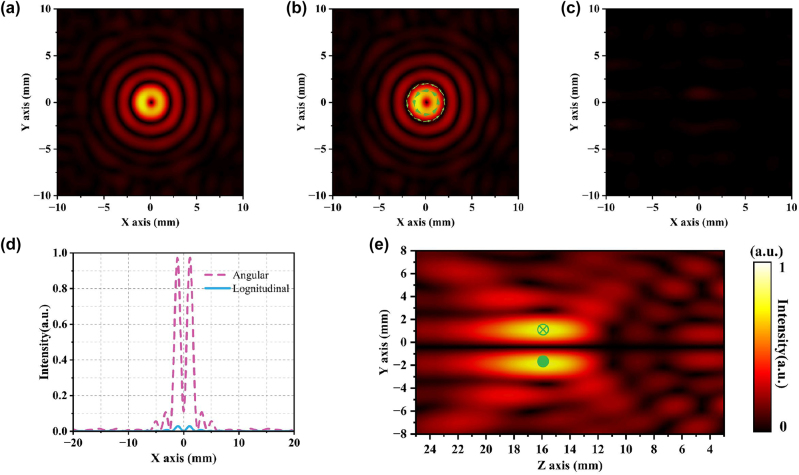
Numerical simulation results of cascaded metamaterial *y*-polarized light: (a) normalized focal plane total field intensity distribution. (b) Normalized focal plane angular field distribution and the polarization distribution at the focal point. (c) Normalized longitudinal field distribution of the focal plane and polarization distribution at the focal point. (d) Normalized light intensity distribution curve of the focal plane along the *x*-axis angular field and longitudinal field. (e) Side electric field intensity along the *z*-axis and focal polarization distribution.

After numerical simulation, we can find that two separate modules can realize the functions of focusing and polarization conversion. Next, we have cascaded the two modules and performed numerical simulations to observe the distribution characteristics of the outgoing optical field. First, the *x*-linearly polarized light is incident on the cascaded device to generate a radial vector tightly focused field, and the total field intensity distribution of the electric field is shown in Figure 5(a). Unlike the single-lens analog case, the focal point appears circular. Figure 5(b) shows the electric field intensity distribution and polarization state distribution of the longitudinal component of the radial tight focusing field. It can be seen that the focal point of the longitudinal field component generated by the cascaded device is circular, and the polarization direction at the focal point is parallel to the propagation direction of the wave vector. Figure 5(c) shows the intensity distribution of the radial component, and its polarization state presents the distribution characteristics of a radial vector beam. The light intensity distribution is a hollow circle with an obvious center void. Figure 5(e) is the lateral distribution of the electric field (*y*–*z* plane), and it can be seen that the focal point is located at 16 mm. The energy of the longitudinal component electric field is much higher than that of the radial component electric field (Figure 5(d)), and there is no angular field component, which is consistent with the previous theoretical derivation. Compared with non-cascaded, the discrete light intensity of the metamaterial array is perfectly collected by the high numerical aperture lens, which makes up for the defect of uneven light intensity distribution. The focal diameter of the radially tightly focused field is significantly smaller than that of a single lens. The vector light converted by the metamaterial array enables the lens to break through. When the *y*-line polarized light is incident, the electric field distribution is sampled and analyzed at the focal plane, and the results are shown in Figure 6. The total field distribution (Figure 6(a)) is almost identical to the angular field component distribution (Figure 6(b)), and the distribution of light intensity and polarization state conforms to the distribution characteristics of angular cylindrical vector beams. Figure 6(c) shows that the light intensity of the longitudinal field component is almost 0, and there is no radial field component, which is consistent with the theoretical analysis. Figure 6(d) shows the angular and longitudinal field components. It can be clearly seen that the energy is almost completely concentrated on the ring belt at the focal point, the central light intensity singularity is clear and obvious, and the light intensity distribution is uniform and symmetrical. The lateral distribution is shown in Figure 6(e), the singularity of light intensity always exists, the polarization state distribution at the cross section is parallel to the top and bottom, and the direction is opposite, and the focus position conforms to the design. The numerical simulation results of the devices before and after cascading are in line with the theoretical design expectations, and the design goals can be achieved.

The diffraction efficiency involved in this paper can be obtained by calculating the ratio of the power flow of the beam transformed by the all-dielectric metasurface to the power flow of the incident beam. The power flow is a far-field observable value, defined by the time-averaged radiant flux per unit area (the time-averaged magnitude of the Poynting vector) over configured distances [75], that is

(21) $$P=\left\langle E_{\text{far}}\times H_{\text{far}}\right\rangle avg.$$

The focusing efficiency of a lens can be calculated using the following equation [76]:

(22) $$Eta=\frac{{\left|E_{FWHM*3}\right|}^{2}}{{\left|E_{\text{total}}\right|}^{2}}*100\;\%.$$

where *E*_FWHM*3_ is the energy in an area three times the full width at half maximum of the imaging area, and *E*_total_ is the incident light energy.

According to the above equation, we obtained that the operating efficiency of the metalens module is 0.37, the operating efficiency of the metamaterial module for generating radial vector beams is 0.872, and for generating azimuthal vector beams is 0.876. The operating efficiency for generating tightly focused radial vector beams after cascading is 0.323, and for angular vector beams is 0.324.

## 3 Experiment and analysis

We choose 3D printing technology as the main processing method. The 3D printer we have chosen for this project is the Shape 1+ printer from RAYSHAPE. It uses DLP light curing technology with low peel force. The printed pixel size is 100 μm, the printed layer thickness ranges from 50–300 μm, and the printing tolerance is 50 μm. The printing accuracy and tolerance are sufficient relative to the unit size that we have designed. To discretization the height, we need to set the printing layer height. The layer height is the height of each layer of the printing part. When the layer height is set lower, the printed surface will be smoother, making the actual preparation effect closer to the simulation design. Therefore, we use the highest level of discretization when printing, with a layer height of 50 μm, which will reduce the printing speed, but can ensure the performance of the prepared metamaterial and metasurface. Among the materials supported by this 3D printer, we have chosen red wax, which boasts the best detail performance and has an advantage in printing smooth surfaces. Simulation results have proved that this material can meet the design requirements. In order to correspond to the simulation results, we printed two modules separately, tested them separately and then conducted a cascade test.

**Figure 7:**
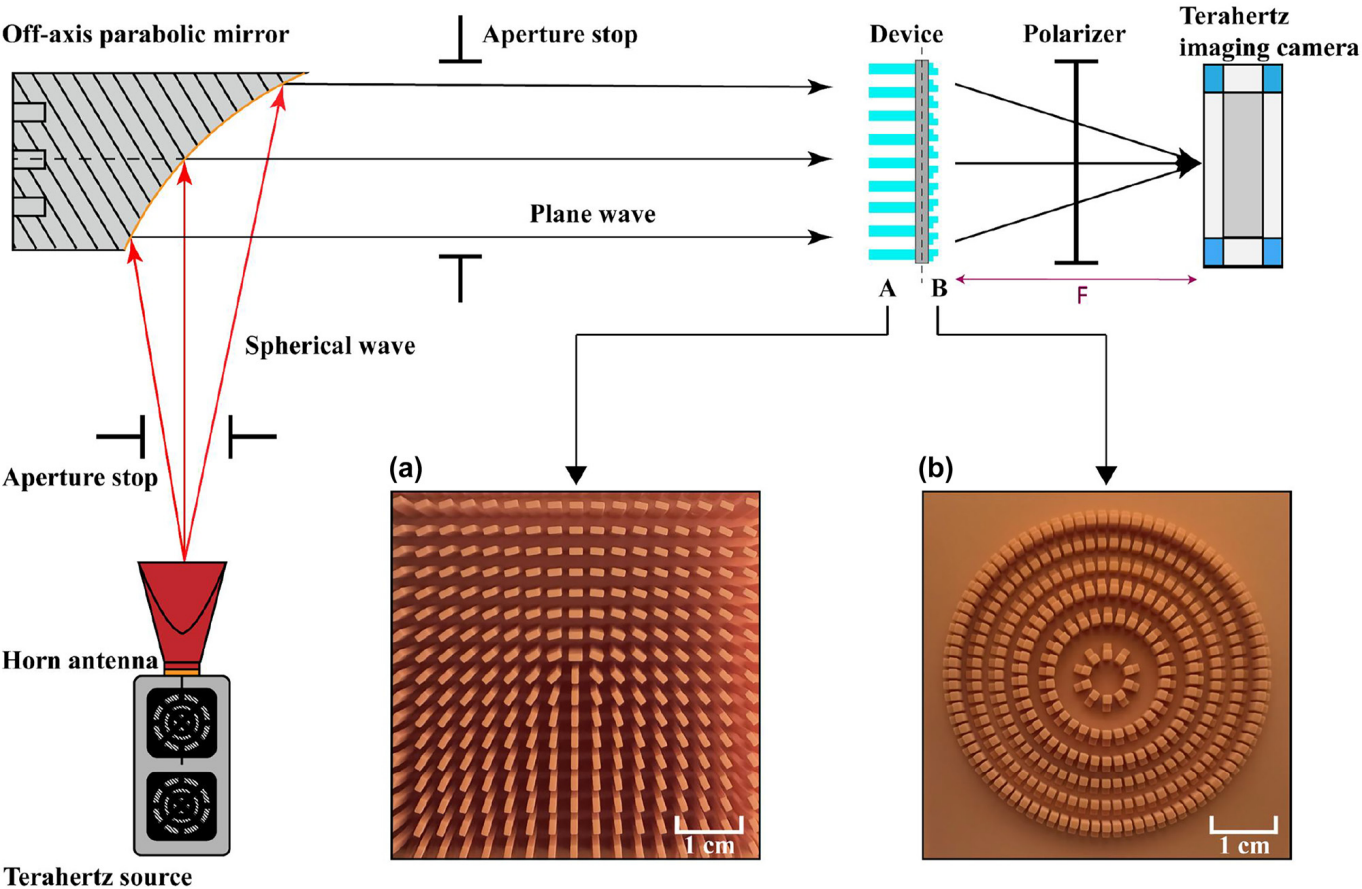
Schematic diagram of the experimental setup. (a) Top view of the metamaterial array sample, and (b) top view of the metalens sample.

The optical path diagram of the experimental test is shown in Figure 7. The light source adopts the terahertz source of TeraSense Company, and the device can work in the frequency range of 0.1–0.3 THz. Connect the terahertz light source with the horn antenna (model Anteral SGH-26-WR10) to transmit the beam. The transmission mode is point source transmission, and the outgoing wave is spherical wave. First, the aperture diaphragm is used to constrain the beam, and then an off-axis parabolic mirror (manufactured by ThorLabs, model MPD369-M01) is set to perform aplanatic processing on the outgoing wave, and the reflected beam still uses the aperture diaphragm to filter out clutter. Irradiating the device we prepared, and at the same time ensure that the distance from the light source to the sample is large enough to ensure the plane wave characteristics of the incident light. The outgoing light converted by the cascade device will be received by the terahertz camera produced by TeraSense, which model is Tera-1024, and the results received by the terahertz camera will be processed on the computer side. The receiving plane is composed of 32 × 32 pixels, each pixel has a side length of 1.5 mm, the working frequency band is 50GHz-0.7 THz, and the high-speed image acquisition rate can reach up to 50 frames per second. Place the terahertz camera at a distance of 16 mm from the sample, and first debug the overall optical path without loading the design module to ensure the stability of the output beam intensity of the light source. Subsequently, the prepared samples were tested, and all test results were normalized.

**Figure 8:**
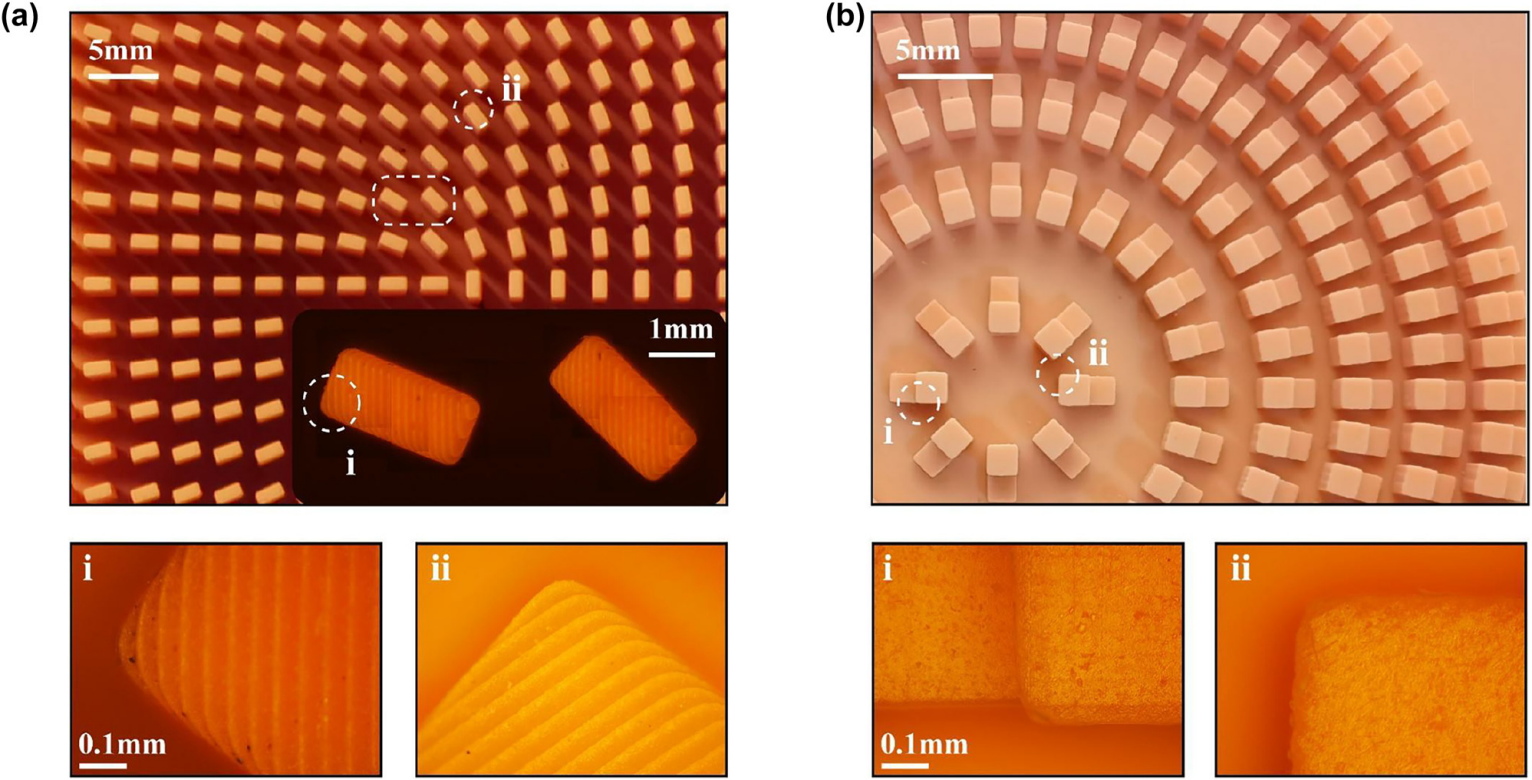
Microscopic image of the module unit structure. (a) Detailed structure diagram of the vector beam generation module. (b) Detailed structure diagram of the metalens module unit.

**Figure 9:**
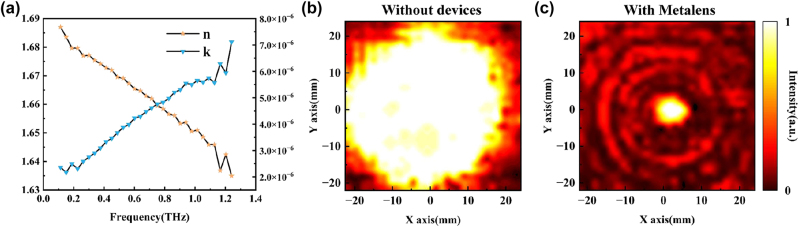
Material parameters for red wax and results of the initial light spot and single lens experiment. (a) Refractive index and extinction coefficient. (b) Intensity distribution of the initial light spot. (c) Intensity distribution of the single metalens experiment.

Figure 8(a) shows the detailed unit of the metamaterial array used to generate vector beams. In the microscopy image, it can be observed that the edges of the rectangular unit are smooth and the rotation angles are in agreement with the design. Figure 8(a) (i)–(ii) show magnified details of the unit at different locations. It can be observed that the distortion of the unit’s corners is very small. Due to the relatively high height of the columns, the overall metasurface is fabricated using inclined printing techniques to avoid deformation of the column structures. As a result, the surface of the structure appears as stripes. The designed height of the metamaterial columns is 16 mm, while the actual average height of the sample was measured as 15.991 mm using a spiral micrometer. By utilizing a microscope, the average length of the long axis of the metamaterial unit cell was measured as 1.956 mm, and the average length of the short axis was 0.984 mm. These dimensions are in excellent agreement with the simulated design values, demonstrating a high level of precision. Figure 8(b) illustrates a detailed microscope image of the metalens module, revealing the average heights of the tall and short columns to be 2.954 mm and 1.496 mm. The average side lengths of the two square columns were measured to be 13.957 mm and 12.951 mm, respectively. Figure 8(b) (i) shows a magnified view of the junction between the two columns in the dual-column structure, where a noticeable difference in size is observed with a protrusion difference of 0.049 mm. Figure 8(b) (ii) depicts a detailed magnified view of the corner of the column structure, where the distortion of the rounded corner is similarly small. As the height of the metalens columns is much lower compared to the metamaterial module, a flat printing method was adopted, resulting in a smooth unit surface without any visible stripes. The experimental test parameters for the material are shown in the Figure 9(a), which serves as a reference for simulation. As can be seen, the material’s losses remain consistently low within the operating bandwidth of the design.

Firstly, we recorded the intensity distribution of the light source without the module, as shown in Figure 9(b). We constrained the light spot to a circle with a radius of 20 mm, enabling us to distinguish the boundaries of the light spot. Integrating the measured light intensity on the camera and subtracting the dark noise yields the integrated intensity of the initial light spot. Subsequently, the working efficiency of each module is similarly obtained by integrating the light intensity on the camera and normalizing it to the recorded integrated intensity when the device is removed. The terahertz camera was placed at the focal point, that is, at a distance of 16 mm from the sample, and a separate lens sample was tested. Figure 9(c) shows the test intensity distribution of the metalens module. At the focal distance, the module generates an elliptical focal spot with a radius of 3 mm. The simulation result shows that the spot radius is 2 mm. We found that limited by the pixel size of the terahertz camera, the gap between the central focus and the secondary light intensity ring in the simulation results is much smaller than the pixel size, which cannot perfectly sample the compact light intensity distribution at the focus. As a result, the actual focus size is slightly larger than the simulation result, but the light intensity ring with a large gap in the outer ring is clear, and produces a good focusing effect, meanwhile the focus is obvious. The module’s working efficiency was measured to be 0.32 through intensity integration.

**Figure 10:**
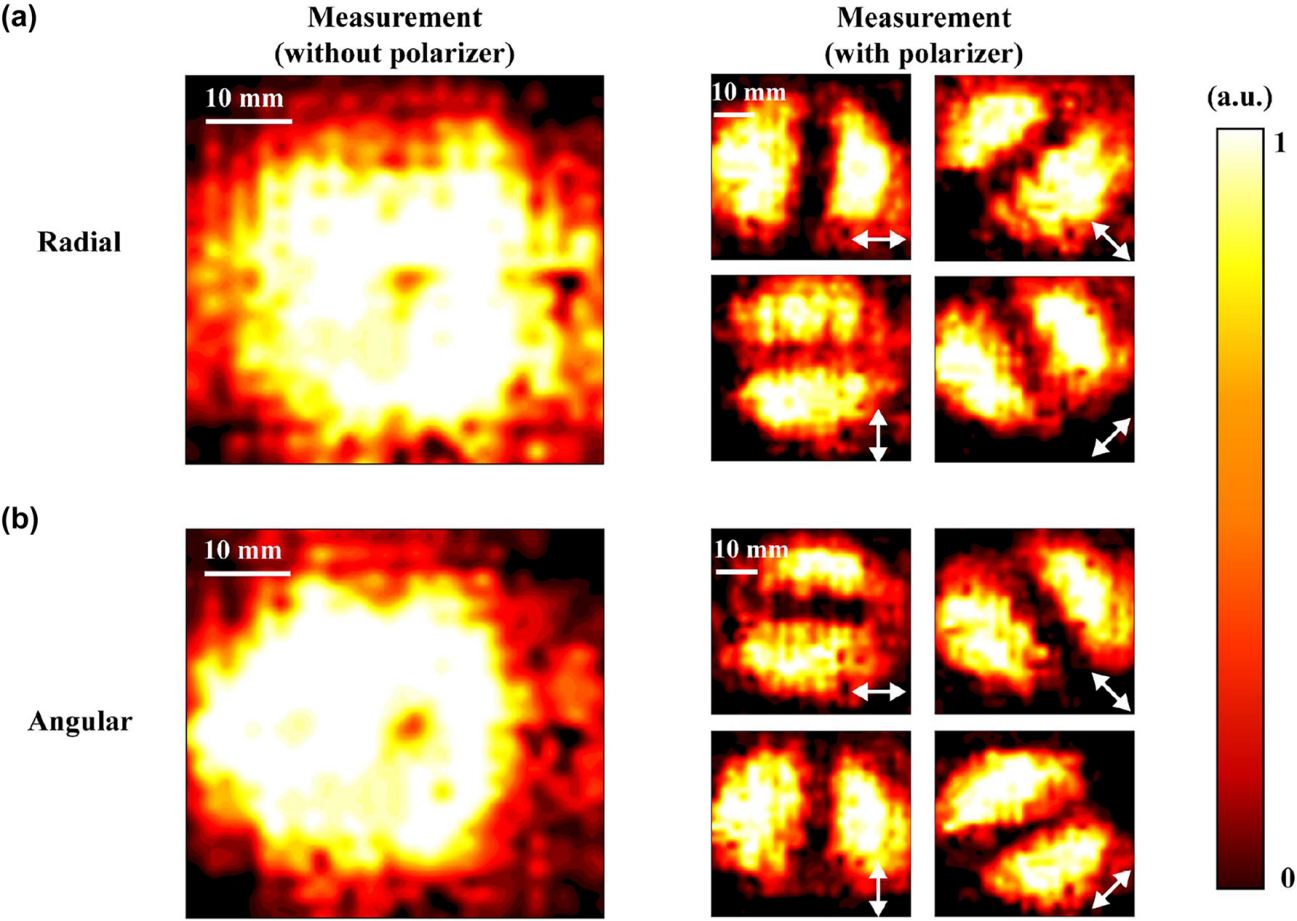
Experimental results of vector light beam generation using metamaterial module. (a) Experimental measurement of the intensity distribution of the radial vector light. (b) Experimental measurement of the intensity distribution of the angular vector beam.

Then, individual metamaterial arrays have been tested. The outermost structural column has a certain deformation due to the thermal deformation effect. We have considered this situation in the design process. The array unit we designed is 19 × 19, and the actual printing array is 21 × 21. The deformation of the outer ring will not affect the experimental results. Place the sample in the optical path to sample the electric field distribution. The terahertz source emits *x*-polarized light, and the metamaterial array converts it into a radial vector beam. Figure 10 presents experimental results of the metamaterial module, the vector characteristics detection results were supplemented by adding a linear polarizer (PW010-025-075) in front of the camera for different vector light generation modes. Figure 10(a) shows the experimental intensity distribution of the radial vector light. It can be observed that the overall spot size without a polarizer still remains around 40 mm, similar to the initial spot. The overall metamaterial transmission is uniform, and a light intensity singularity with a radius of 1.5 mm is generated at the center. The edge intensity of the spot weakens, with the presence of scattered distribution of light intensity, which conforms to the simulation results. After adding a polarizer, in the intensity distribution graph filtered through polarized light, there is a gap at the center with a width of about 4.5 mm, which rotates with the rotation of the polarizer.

When the linear polarizer is aligned with the *x*-axis, the gap perpendicular to the *x*-axis. By rotating the polarizer clockwise 45°, the gap rotates counterclockwise 45°. Then, as the polarizer is rotated to 90° and 135°, the dark stripes still align with the polarization characteristics of radial vector beams. Figure 10(b) depicts the experimental result of the angular vector light, where the size of the spot is slightly smaller than that of the radial vector light mode when no polarizer is placed. Similarly, tests were conducted on angular vector beams, and at the same polarizer rotation angles, the direction of the gap is orthogonal to that of radial vector beams, as shown in Figure 10(b), which conforms to the polarization characteristics of angular vector beams. By comparing with the initial spot through integration, the efficiency of generating radial vector light in this module is 0.73, while the efficiency of generating angular vector light is 0.71.

**Figure 11:**
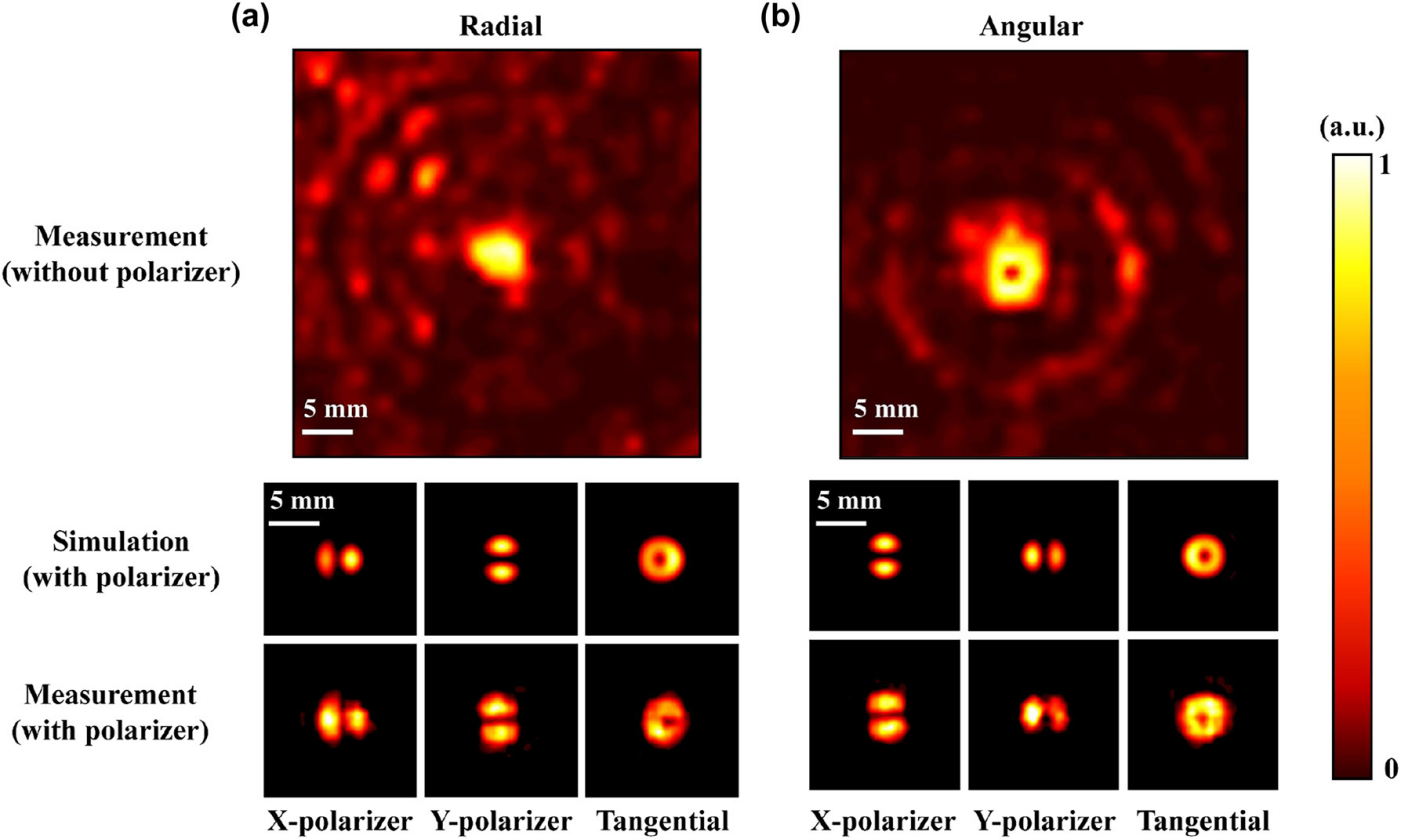
Experimental results of cascaded metamaterials. (a) Intensity distribution of tightly focused experimental radial vector beam. (b) Intensity distribution of tightly focused experimental azimuthal vector beam.

The substrates of the two modules are the same size, and the side lengths are slightly larger than the simulation size. While ensuring the integrity of the working area, the extra side length is convenient for cascading and fixing in the optical path. Fasten the two individual modules on four sides with a clamp to construct a cascaded device sample. As is shown in the Figure 11, the spot radius of the tightly focused radial vector beam mode was measured as 2 mm, and that of the angular vector beam mode was 2.25 mm, both in agreement with the simulation results. Figure 11(a) shows the normalized experimental intensity distribution of radially polarized vector beams after tight focusing. The polarizing filtering results aligned with the *X* and *Y* axes still conform to the polarization characteristics of radially polarized vector beams, and are consistent with the simulation results. As shown in the Figure 11(a), its tangential component is an annular spot with a central intensity singularity, and the total field is a solid spot. The central intensity is significantly stronger than that at the edge of the spot, indicating the presence of a strong longitudinal component generated by tight focusing. Figure 11(b) shows the testing result of angular vector beams. The normalized intensity distribution after polarizing filtering also conforms to its angular polarization characteristics. The tangential field component is a hollow annular spot, consistent with the total field intensity. Only by changing the polarization direction of the incident beam, the solid focus was transformed into a hollow focus, which is enough to prove that the structure we designed can achieve the expected function. The experimental results are basically consistent with the simulation results.

## 4 Conclusions

We have designed a metamaterial array capable of converting orthogonal linearly polarized incident light into corresponding cylindrical vector beams. Combined with another high numerical aperture metalens module designed, the two were physically cascaded to generate a vector beam tight focus field, which was theoretically analyzed and numerically simulated, and the preparation and experimental verification were carried out. Based on the high degree of freedom of microstructure fabrication by 3D printing technology, we were able to use cascaded devices to achieve tightly focused field generation. At a frequency of 0.1 THz, we tested the two independent modules and the cascaded modules in experiments. The experimental results are basically consistent with the simulation results. Our research provides a new idea for the regulation of tightly focused fields in the terahertz band.
